# Targeted memory reactivation of face-name learning depends on ample and undisturbed slow-wave sleep

**DOI:** 10.1038/s41539-021-00119-2

**Published:** 2022-01-12

**Authors:** Nathan W. Whitmore, Adrianna M. Bassard, Ken A. Paller

**Affiliations:** Department of Psychology, Cognitive Neuroscience Program, and Interdepartmental Neuroscience Program, Northwestern University, Evanston, IL 60208-2710 USA

**Keywords:** Long-term memory, Human behaviour

## Abstract

Face memory, including the ability to recall a person’s name, is of major importance in social contexts. Like many other memory functions, it may rely on sleep. We investigated whether targeted memory reactivation during sleep could improve associative and perceptual aspects of face memory. Participants studied 80 face-name pairs, and then a subset of spoken names with associated background music was presented unobtrusively during a daytime nap. This manipulation preferentially improved name recall and face recognition for those reactivated face-name pairs, as modulated by two factors related to sleep quality; memory benefits were positively correlated with the duration of stage N3 sleep (slow-wave sleep) and negatively correlated with measures of sleep disruption. We conclude that (a) reactivation of specific face-name memories during sleep can strengthen these associations and the constituent memories, and that (b) the effectiveness of this reactivation depends on uninterrupted N3 sleep.

## Introduction

We often rely on face recognition and name recall—such as when we notice friends from a distance and call to them by their names. Most people are extraordinarily adept at recognizing the faces of individuals, even those they’ve met just once. Yet, there are also times when we fail to recognize someone—and it can be embarrassing when we forget a name that we should have remembered.

What determines which memories continue to be enduringly available and which are forgotten? Given that the human brain is remarkably active during sleep, researchers have asserted that neural events during sleep may function to stabilize and strengthen recently acquired memories^1–4^. The delineation of these neural events and their specific ramifications for memory has become increasingly central to memory research and the science of learning.

A prevalent view is that memories can benefit due to spontaneous replay during sleep^5–8^. In recent years, Targeted Memory Reactivation (TMR) has emerged as a useful tool for investigating this process^9^. In the TMR procedure, information that people learn is associated with a sound or smell during learning. Researchers then present the same sensory cue, while people sleep, without waking them. After sleep, people remember information associated with the cue stimulus better than other information that was equally well-learned, a frequently reported finding confirmed in a recent meta-analysis^10^. Moreover, the notion that TMR benefits memory through reactivation is supported by neuronal evidence of hippocampal place cell replay engaged following the presentation of learning-related sounds during sleep^11^.

In addition to functioning as a powerful research tool, TMR offers the potential to enhance memory with a simple, noninvasive intervention during sleep, which may be useful in many scenarios. Because learning face-name associations is an important and widely relevant form of memory, we asked whether TMR could enhance this type of learning.

**Fig. 1:**
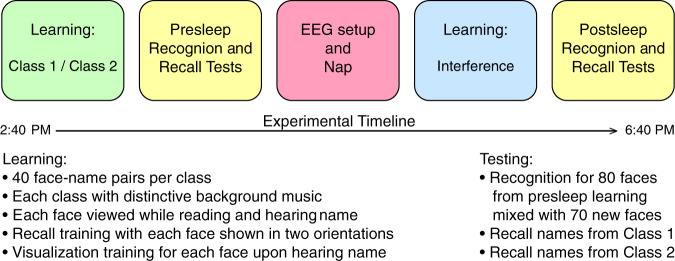
Experimental procedure overview. Stages of the protocol are illustrated at the top (with mean beginning and ending times). Details of the learning and testing procedures are listed below.

We developed a procedure whereby participants learned about people ostensibly in either a Japanese History class or a Latin-American History class. Learning was accompanied by a background music track, either traditional Japanese music or traditional Latin-American music, respectively. Each classroom had 40 pupils. To learn the names of these pupils, participants viewed each face adjacent to the corresponding written name, while also hearing the spoken name. Recall training, feedback, and visualization practice served to solidify this learning. Next, we assessed both face recognition and name recall. Then, during a period of sleep, some of the spoken names and associated background music were softly presented. After awakening, participants were exposed to additional faces and names, potentially interfering with the original learning. Finally, we assessed memory again. Figure 1 shows the experimental procedure schematically, and additional details are provided in METHODS.

This experimental design thus makes it possible to determine the extent to which face memory can be selectively improved by reactivation during sleep. Such results, along with analyses of relationships between memory change and characteristics of slow-wave sleep, could have implications for understanding the physiological mechanisms that generally enable memory reactivation during sleep to be effective.

## Results

### The influence of TMR on memory varied with duration of stage N3 sleep

Memory testing demonstrated effective learning of faces and associated names. On the name recall test, participants correctly recalled a mean of 74.04 (SD = 4.83) names before sleep and 75.00 (SD = 4.03) names after sleep (in each case from a total of 80 face-name associations). When requested, hints were provided in the form of up to three starting letters. Across all 80 names in the test, participants requested a mean of 0.77 (SD = 0.69) hint letters per name before sleep and 0.80 (SD = 0.75) after sleep. On the presleep recognition test, participants successfully recognized 97% (SD = 3%) of the identical old faces and 92% (SD = 7%) of the old faces that were rotated from their original view. For new faces, 74% (SD = 24%) were endorsed correctly as new faces. Postsleep recognition scores were similar (97%, 91%, and 85% correct for old, rotated, and new, SDs = 4%, 9%, 15%, respectively).

To examine the effect of TMR, we first computed the change in the number of names successfully recalled across sleep. This value, Δrecall, did not differ significantly between the cued class, designated *class C*, and the uncued class, designated *class U* [mean Δrecall 0.75 and 0.21, SDs = 2.03 and 1.70, respectively; *t*(23) = 0.93, *p* = 0.36].

**Fig. 2:**
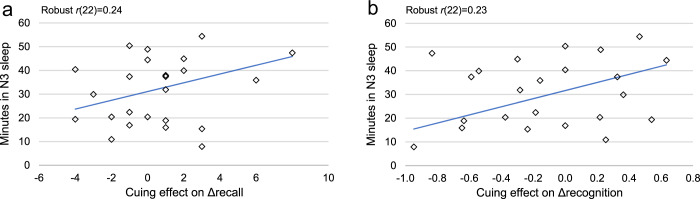
Correlations between N3 duration and cueing effect. N3 duration was correlated with the cuing effect on Δrecall (**a**), and with the cuing effect on Δrecognition (**b**).

In initial planned comparisons, we analyzed the cueing effect on *Δrecall* (defined as the difference between class-C Δrecall and class-U Δrecall) in relation to several sleep measures: total sleep duration, N2 duration, N3 duration, and REM duration. As shown in Fig. 2a, the size of the cuing effect on Δrecall was correlated with the duration of stage N3 sleep [*χ²* (1*, N* = 24) = 5.18, *p* = 0.02]. This cueing effect was not significantly correlated with the duration of other sleep stages. Taking into account the total sleep duration during the nap, a follow-up exploratory analysis revealed an even stronger association between the cueing effect and relative duration of N3 sleep [*χ²* (1*, N* = 24) = 14.52, *p* < 0.001, FDR *p* = 0.001].

We found similar effects with face recognition. To measure recognition accuracy, we collapsed performance across old and rotated faces and computed d’ statistics for class C and class U before and after sleep. Overall, recognition accuracy increased after sleep [mean increase = 0.43, SD = 0.47, t(22) = 4.37, *p* < 0.001], but Δrecognition (change in d’) did not differ between class C and class U [mean Δrecognition 0.37 and 0.49, SD = 0.50 and 0.54, respectively; *t*(22) = 1.25, *p* = 0.22]. Nevertheless, the cuing effect on Δrecognition was associated with N3 duration (Fig. 2b), paralleling the results for name recall [*χ²* (1*, N* = 23) = 5.56, *p* = 0.02].

### The influence of TMR on recall depended on undisturbed sleep

A previous report found that participants who self-reported sleep disruption during TMR did not benefit from memory cues^12^. To objectively assess sleep disruption related to cue delivery during sleep, we developed the Sleep Disruption Index to quantify arousals in the EEG after spoken name cues. As an arousal is conventionally defined by an abrupt shift in the spectral content of the EEG^13^, we summed the absolute change in EEG power at Cz (increase or decrease) across a broad frequency band from 0.38 to 20.35 Hz during the 5 seconds after spoken-name cue onset relative to the 5 prior seconds. The Sleep Disruption Index thus focuses on periods surrounding name cue presentation. Additionally, we computed another measure that encompasses the whole nap period, the Sleep Fragmentation Index, which quantifies sleep-stage transitions per hour^14^. We then used a correlational analysis to examine whether the memory benefit of TMR was associated with these indices of sleep disruption.

**Fig. 3:**
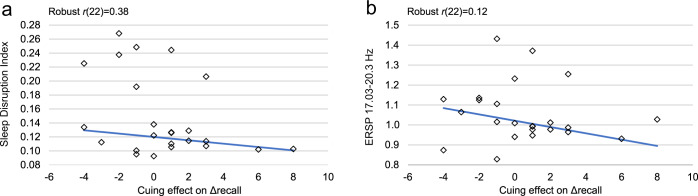
Correlations between sleep disruption and cueing effect. Sleep disruption after a cue was negatively correlated with the cuing effect on Δrecall (**a**﻿). Greater post-cue EEG power in the high beta band (17.03-20.35 Hz) was associated with a smaller cuing effect (**b**).

As shown in Fig. 3a, cue-related sleep disruption, as assessed by the Sleep Disruption Index, was negatively correlated with the cuing effect on Δrecall [*χ²* (1*, N* = 24) = 3.98, *p* = 0.046]. There was a trend for a similar effect with the Sleep Fragmentation Index [*χ²* (1*, N* = 24) = 2.57, *p* = 0.109]. We did not observe correlations between either sleep index and recognition performance.

Because the Sleep Disruption Index encompasses a broad frequency range, we conducted follow-up analyses to identify whether certain frequency bands were associated with the cuing effect on Δrecall. We divided the 0.38–20.35 Hz spectrum into six frequency bands, each 3.33-Hz wide, and then we correlated cue-evoked power in each band with the cuing effect on Δrecall. Cue-evoked power in the highest beta band (17.03–20.35 Hz) was negatively correlated with the cuing effect; a greater cuing effect was seen with less beta power after a cue [*χ²* (1*, N* = 24) = 10.1, *p* = 0.0015, Fig. 3b]. We also found marginal negative correlations between the cuing effect on Δrecall and power in the lower alpha band [7.04–10.37 Hz, *χ²* (1*, N* = 24) = 3.83, *p* = 0.05] as well as power in a lower beta band [13.7–17.03 Hz, *χ²* (1*, N* = 24) = 3.43, *p* = 0.06]. As abrupt increases in alpha and beta are associated with arousal from sleep^13^, these data suggest that TMR depends on uninterrupted sleep.

### Measures of N3 duration, cuing, and sleep disruption were highly intercorrelated

N3 duration was highly correlated with the Sleep Disruption Index [*r*(22) = −0.6, *p* = 0.002], Sleep Fragmentation Index [*r*(22) = −0.65, *p* = 0.001], number of name cues presented during sleep [*r*(22) = 0.59, *p* = 0.002], and total number of sleep-stage transitions [*r*(22) = −0.44, *p* = 0.03]. To test whether these variables independently explained variance in the cueing effect on Δrecall, we performed multiple regression. The overall regression model predicted the cuing effect on Δrecall [*r*(17) = 0.55, *p* = 0.01]. Several variables had significant coefficients: Sleep Fragmentation Index [*χ²* (1*, N* = 24) = 6.39, *p* = 0.01], Sleep Disruption Index [*χ²* (1*, N* = 24) = 6.23, *p* = 0.01], and number of name cues [*χ²* (1*, N* = 24) = 5.23, *p* = 0.02]. Therefore, these measures represent correlated but separable facets of overall sleep quality.

### Additional exploratory analyses

We also performed correlations between cuing effects and several other sleep and participant variables, as described in Supplemental Tables 1 and 2. Notably, in addition to the correlations with time in N3, the cuing effect on Δrecall was positively associated with participant age and percent of N3 sleep during the nap. The cuing effect on Δrecall was negatively associated with total sleep-stage transitions during the nap, which is a component of the Sleep Fragmentation Index. In addition to the association with N3 time, the cuing effect on Δrecognition was negatively associated with measures of stage 2 sleep (percent stage 2 sleep and sigma power) as well as with participant age. We also correlated N3 time and sleep disruption with other memory measures (Supplemental Table 3). The finding that older participants showed a larger cuing effect may have reflected higher sleep quality in these individuals; these older participants (the oldest of which was 31 years old) had a lower Sleep Fragmentation Index [*χ²* (1*, N* = 24) = 4.08, *p* = 0.04] and a lower Sleep Disruption Index [*χ²* (1*, N* = 24) = 4.69, *p* = 0.03], and they received a larger number of spoken cues [*χ²* (1*, N* = 24) = 4.79, *p* = 0.03].

We used a decision-tree partition analysis (JMP 15) to ask whether we could classify participants as TMR responders or non-responders based on number of spoken cues, N3 duration, or sleep disruption index. Sleep Disruption Index was the single strongest predictor of the Δrecall cuing effect. Participants with a Sleep Disruption Index < 0.13 (*n* = 15) had a positive Δrecall cuing effect [mean = 1.53 names, SD = 2.75, *t*(14) = 2.16, *p* = 0.049], indicating superior recall for the cued class. Participants with a Sleep Disruption Index > 0.13 did not have a significant Δrecall cuing effect. For the cuing effect on Δrecognition, number of spoken cues was the single strongest predictor; participants with >157 spoken cues had a nonsignificant trend for a positive cuing effect [mean = 0.37, SD = 0.27, *t*(3) = 2.73, *p* = 0.072], whereas those with fewer spoken cues had a negative cuing effect [mean = −0.22, SD = 0.42, *t*(18) = 2.29, *p* = 0.03].

## Discussion

In this experiment, we observed that presenting cues during sleep influenced two important memory abilities: recognizing a face that was recently viewed and recalling a person’s name when seeing their face. To the extent that slow-wave sleep was ample and undisturbed, cues reactivated recent memories during sleep and thereby improved both the ability to recognize faces and the ability to recall the associated names. We conclude that memory reactivation during sleep can improve memory for people’s faces and names. Notably, benefits of memory reactivation were greater in those individuals with longer periods of slow-wave sleep and without signs of sleep disruption during their afternoon nap in the laboratory.

Delivering auditory or olfactory cues during slow-wave sleep with the goal of targeted memory reactivation has previously been shown to improve many types of memory^10^. The conclusion that memory reactivation during sleep supports memory function has received support from these studies conducted in many countries, during both afternoon and nocturnal sleep, and in both laboratory and home environments. In this research area, however, learning of face-name associations has not previously been examined. Our results add to this literature by showing that these additional types of memory, name recall and face recognition, are subject to memory reactivation during sleep. The experiment also provided novel evidence that might be generally applicable with respect to any type of learning. That is, objective measures of N3 duration and sleep disruption after cues moderated the degree to which memory reactivation during sleep produced name-recall improvement.

Compatible findings have been reported from studies of TMR in word-learning paradigms using self-reports of sleep disruption^12^ and using measures of REM duration^15^. We propose that variation in quality and quantity of sleep represent important factors sometimes overlooked in the broader TMR literature.

Such variation may explain some instances in which TMR does not benefit memory. Meta-analysis has shown that the extant use of TMR methods produced a significant, small-to-moderate effect size overall, but the effect size was highly variable even across similar studies^10^. Differences in sleep quality and disruption may explain some of this variability; these parameters are not usually reported. Moreover, our results suggest that optimizing sleep quality during TMR may increase the overall size of TMR benefits on memory or other aspects of cognition.

Our results also raise a further question: what is the relationship between momentary arousal, N3 duration, and TMR effects? Göldi and Rasch^12^ proposed that sleep disruption caused by auditory stimulation may introduce interference into newly reactivated memories. Conversely, arousals may have no direct effect on memory, but simply reduce N3 duration, which in turn reduces TMR benefits. A third possibility is that short N3 duration and high arousability both reflect a latent factor of sleep quality that mediates TMR effects, as suggested by the inter-correlations between measures of arousal and N3.

Whereas N3 duration was associated with cuing effects in both the recognition and recall tests, we observed several differences between the tests; for instance, sleep disruption was associated with cuing effects in the recall test but not in the recognition test. The correlations between participant variables, such as sleep parameters and the cuing effect on memory may be shaped by several factors that differ between the tests, including ceiling effects, the total amount of forgetting, and unintended spreading of reactivation from the cued class to the uncued class. There are multiple ways to quantify memory performance (e.g., as shown in Supplemental Table 1), and measures vary in sensitivity and other psychometric properties. Modified protocols, for instance with a between-subjects design or memory testing at a longer delay, may shed light on the role of these factors. When memory is tested immediately after sleep with TMR, evidence that reactivation was helpful might be most clearly evident for memories that would otherwise fall just short of being strong enough to be remembered at that time point.

In our procedure, sleep cues included both specific spoken names from the learning phase, as well as the background track for learning, which was a unique music genre. We therefore cannot determine whether TMR benefits were due to the spoken names, the music, or both. In prior experiments, TMR benefits have been observed using spoken words^16,17^, as well as short music tracks^18,19^.

Other limitations of this study could also be addressed in future research. In particular, it was not possible to measure how sleep disruption after cues affected the fate of individual memory items. Future studies that take into account item-specific sleep disruption measures may shed more light on mechanisms of memory change. Finally, because we recorded only one sleep session, it is unclear whether differences in sleep quality represent stable individual differences. Future studies could employ additional manipulations that address such questions.

Overall, the present results show that provoking memory reactivation during sleep can influence learning of face-name associations, with consequences for whether people can recall the correct name after sleep. Furthermore, the magnitude of this effect was found to depend on the length of N3 sleep and on the absence of signs of arousal after spoken cues. We propose that N3 duration and cue-evoked arousal are important factors shaping how memory reactivation during sleep influences subsequent memory performance. These factors thus have relevance for future research aimed at making progress in understanding the neural mechanisms whereby learning is influenced by subsequent sleep even when there is no sensory input during sleep. Considerations of N3 disruption might be especially relevant when memory modification is desirable, including in clinical contexts^20^. Measures of sleep disturbance should thus be examined closely in future studies seeking to determine if applications of targeted memory reactivation can produce clinical or other benefits.

## Methods

### Participants and Experimental Design

Participants (*N* = 24) were 8 males and 16 females 18–31 years old (mean = 23.38, SD = 4.44) recruited from the Northwestern University population and surrounding community. Figure 1 shows an overview of the procedure, which was approved by the Northwestern University institutional review board. The design provided within-subject comparisons of memory performance as a function of whether cues were presented during sleep. To increase the chance of napping, participants were asked to go to bed 1 h later or wake up 1 h earlier than normal, and to avoid nicotine or caffeine on the day of the study. After participants arrived in the lab and gave written informed consent, the following phases transpired: learning, pre-nap tests, bioelectric recording setup, nap, interference learning, and post-nap tests. After completing these steps, they were paid for their participation.

### Procedure

#### Learning phase

Participants completed a face-name learning task intended to simulate learning the names of pupils in two classes, with 40 face-name pairs per class. Each class was associated with a distinct instrumental music track, traditional Latin-American music or traditional Japanese music. Participants completed all learning tasks for Class 1 followed by all learning tasks for Class 2.

Face-name learning started with initial exposure plus interspersed name recall. Participants viewed sets of five face-name pairs, each shown one at a time while the spoken name was played over speakers. In addition to the name, which included a first name and a surname, a one-sentence fact about the person (such as “I love cats and fall weather”) was also viewed. Each to-be-learned person was presented twice during initial exposure with the same biographical information, with the face shown once in a quarter-right and once in a quarter-left orientation, to encourage learning of facial structure. Prompts for name recall appeared after every set of five pairs; each of the five faces appeared and participants attempted to type the corresponding first name. Following each recall attempt, feedback was provided (the face paired with the correct name simultaneously shown and spoken).

Following initial exposure with interspersed name recall for all 40 pupils in the class, participants began recall training. In this task, the same faces (both orientations) were shown, one at a time, as prompts for recall of the corresponding first name. When a correct name was recalled and entered via the keyboard, the facial image was dropped from the training list. The remaining facial images were shown repeatedly, in random order. Visual and auditory feedback was again provided on each trial. The training was complete when the participant gave the correct first name for each person in the class on two occasions (i.e., after seeing both facial orientations).

Participants were then asked to visualize each person in the class when the corresponding name was spoken. This task was intended to promote face visualization in response to names, even with names presented during sleep. Each visualization attempt was followed by a prompt to identify the visualized person from two same-sex alternatives, using a foil face randomly chosen from the same class as the visualized face. The two faces were presented 3.5 s after name onset. Feedback was given after the participant made each choice (the face paired with the spoken and written name).

Following training for Class 1, all training steps were repeated for Class 2, with the order of the Japanese History class and the Latin-American History class counterbalancing across subjects. Participants completed all learning steps for both classes in approximately 45 min.

#### Presleep and postsleep tests

The first test given was a self-paced recognition test for faces. Participants viewed 230 faces sequentially and were asked to decide if each person was in Class 1, Class 2, or was a new person not seen before. They were asked to perform this categorization regardless of whether the face was rotated from the view seen earlier. Each test included the 80 previously learned faces identical to the images seen earlier (*old*), 70 new faces (*new*), and each of the 80 previously learned faces from a different angle (*rotated*). Old faces were presented at quarter-right orientation, whereas rotated faces were profile views, facing right in the presleep test and left in the postsleep test. New faces were presented with equal numbers at quarter-right orientation and the rotated orientation for that test (profile left or profile right). New faces in both recognition tests were faces not shown previously. Faces were presented in random order. Due to a technical issue, recognition data were not available for one participant.

Name recall followed recognition testing. Participants were asked to type a person’s first name given a picture of the corresponding face. Faces appeared in either a quarter-right or quarter-left orientation one at a time in random order. Participants could receive hints by pressing the tab button to receive a letter, up to the first three letters of the person’s first name. No feedback was provided during this test. Each participant performed a cued recall test for Class 1 and then for Class 2.

The same recognition and recall tests were also given starting approximately 10 min after the end of the sleep phase, immediately following an interference task. In the interference task, participants were asked to memorize 20 new face/name pairs in a simplified procedure with no recall step or biographical details. This manipulation was intended to increase difficulty and simulate the type of interference that occurs in real-world face-name learning.

#### Physiological recording

We recorded electroencephalogram (EEG), electrooculogram (EOG), and electromyogram (EMG) signals during the nap using a BioSemi Active2 system with 32 scalp channels and 4 electrodes on the face. Data were acquired with a sampling rate of 512 Hz, filtered between 0.1 and 100 Hz during recording, and re-referenced to the right mastoid. EOG was recorded with electrodes lateral to the left and right outer canthus and underneath the right eye. EMG was recorded from the chin. Setup and recording began immediately before the start of the sleep period and electrodes were removed after sleep and before the interference task.

#### Nap and TMR

After EEG setup, participants slept on a futon in the same chamber where they completed the learning and testing tasks, with background white noise at a low level (43–44 dB). Participants slept for a mean of 59 min (range: 32–92 min). TMR began after participants had been asleep for a mean of 7.3 min (range: 1.6–21 min). TMR was manually initiated when the experimenter visually detected signs of stage N3, which has characteristic EEG slow waves. TMR was paused when sleep transitioned to any other sleep stage or to wake, and resumed when participants re-entered N3. Offline, raters blind to when cuing occurred determined sleep stages according to standard rules for adults^13^.

The specific TMR cues presented were selected as follows. The class randomly assigned to be cued was Class 1 for half of the subjects and Class 2 for the other half. There was thus a cued class and an uncued class (*Class C* and *Class U*, respectively). During TMR, the background music presented while learning class C was played continuously at low volume and half of the spoken names from Class C were presented at 10-s intervals. The specific names played during sleep were chosen to match presleep recall accuracy and number of hints required between cued and uncued names in class C. Intensity of the spoken names was controlled manually to deliver cues at the highest volume possible without causing arousal (45–50 dB peak). This design was chosen to reactivate both the general context of the cued class and specific items in this class, as both strategies may promote memory benefits^16,18,19^.

### Behavioral data analysis

To test whether cuing differentially influenced memory, we compared the change in memory performance across sleep for class C to that for class U. We defined the *cuing effect* by taking the difference between the two change scores as follows: (class C postsleep—class C presleep)—(class U postsleep—class U presleep). Statistical significance was assessed using a two-tailed *t* test. Due to a technical error, we were not able to test whether TMR effects differed for the two conditions within class C (cuing with spoken name+background music and cuing with only background music).

To measure memory performance in the name recall test, we counted the number of faces for which the participant correctly entered the first name (with or without hints) in each class. To measure recognition performance, we computed a d’ statistic with log-linear correction^21^ for each class based on the hit rate for that class and the false alarm rate pooled across both classes. Recognition statistics were computed treating both rotated and old faces as “old.”

Because participants designated each face as either “class 1”, “class 2”, or “new” during the recognition test, we considered either a “class 1” or “class 2” response as an “old” response, independent of whether the participant attributed the old face to the correct class.

### Arousal analysis

Arousal during sleep is conventionally defined as an abrupt shift in the spectrum of the EEG^13^. Therefore, we quantified arousal after TMR cues by measuring the absolute difference of the power spectra in two windows: [−5 0] seconds relative to cue onset and [0 5] seconds relative to cue onset. Arousal was calculated as the mean of abs(1-(postcue power/ precue power)) across linear-spaced 0.256-Hz-wide frequency bins from 0.38 to 20.35 Hz. Power was calculated using a short-time FFT (newtimef, EEGLAB 14.1.1b, 2-s window)^22^. These parameters were chosen to cover the range of frequencies in which arousal-related activity appears, while providing sufficient frequency and time resolution to compute the sleep disruption index. As a secondary analysis to identify the frequencies correlated with TMR effects, we divided this frequency range into six 3.33-Hz-wide bins and correlated the event-related spectral-power change for each bin (postcue power/precue power) with the cuing effect.

### Correlation analysis

Because we observed outliers and heteroskedasticity in many of our correlates (especially higher variance in sleep measures when the cuing effect was low), we computed correlations between the cuing effect and sleep variables using robust linear regression. These regressions were performed with a Cauchy error distribution as implemented in JMP 15. Robust regressions were calculated with cuing effect as the independent variable and sleep measures as the dependent variable. Significance was determined using a Wald test to ascertain whether including the cuing effect significantly improved the prediction of the dependent variable. Robust *r* values correspond to the variance in the unweighted data explained by the Cauchy regression line. We also computed correlations using ordinary least squares, which gave similar results (Supplemental Tables 3 and 4). Intercorrelations between sleep measures were computed using ordinary least squares as these correlations were not heteroskedastic. P values were adjusted for multiple comparisons using the false discovery rate^23^.

### Reporting summary

Further information on research design is available in the Nature Research Reporting Summary linked to this article.

## Supplementary information

Supplemental Information

Reporting Summary

## Data Availability

The data that support the findings of this study are available from the corresponding author upon reasonable request.

## Code availability

Custom software developed for this study is available from the corresponding author upon reasonable request.
