# Supplementary material for: Targeted memory reactivation of face-name learning depends on ample and undisturbed slow-wave sleep
Source: NPJ Sci Learn. 2022 Jan 12;7:1. doi: 10.1038/s41539-021-00119-2 (PMC8755782; doi:10.1038/s41539-021-00119-2)
Supplement: Supplementary file 1 — Supplemental Information [file 41539_2021_119_MOESM1_ESM.pdf]

Supplemental Information

Targeted Memory Reactivation of Face-Name Learning Depends on Ample and Undisturbed Slow-Wave Sleep

Nathan W. Whitmore, Adrianna M. Bassard, and Ken A. Paller
Northwestern University

Supplemental Table 1: Participant and sleep metrics and their correlation with cuing effects as measured by Δrecall. Stage transitions and sleep fragmentation index were calculated as described in Haba-Rubio, Ibanez, and Sforza (2004). SO (0.5-1 Hz), delta (1-4 Hz), and sigma (12-16 Hz) power were calculated between the first and last sleep epoch of recordings using short-time FFT (spectopo, EEGLAB 14.1.1b, 2-s window) after removing time periods containing artifacts. Bold text indicates significant correlations. Percent sleep times were calculated as the proportion of total sleep time spent in a specific phase. Sleep disruption index was calculated as described in the methods. We also calculated correlations using ordinary linear regression (OLS) which found a similar but less robust pattern due to outliers and heteroskedasticity in the data. Bold font indicates statistical significance.

Measure	Mean	*SD*	Cauchy chi^2	Cauchy *p*	Cauchy *p* (FDR)	Cauchy r	OLS r	OLS *p*
Total sleep min	59.04	15.34	2.2	0.14	0.25	-0.44	-0.08	0.71
Stage 2 min	19.17	13.12	1.13	0.29	0.45	-0.61	-0.13	0.54
**Stage 3 min**	**31.33**	**13.82**	**5.18**	**0.02**	0.07	1.15	0.24	0.26
REM min	1.48	4.68	0.00	0.99	1.00	-0.66	-0.40	0.05
**Age** (years)	**23.38**	**4.44**	**31.45**	**<0.001**	**<0.001**	**0.68**	**0.44**	**0.03**
**Number of name cues**	**118.63**	**41.19**	**5.67**	**0.02**	0.07	3.97	0.27	0.20
Min between tests	113.42	14.82	2.06	0.15	0.26	-54.02	-0.17	0.42
Min sleep after last cue	35.66	14.49	0.92	0.34	0.48	-73.01	-0.24	0.26
**Total stage transitions**	**23.63**	**11.20**	**8.05**	**0.005**	**0.03**	-1.46	-0.37	0.07
Sleep fragmentation index	24.81	13.17	2.57	0.11	0.26	-1.63	-0.35	0.09
Slow oscillation power (μV^2)	515.14	358.31	2.07	0.15	0.26	15.94	0.13	0.56
Delta power (μV^2)	117.88	89.28	0.24	0.62	0.7	0.97	0.03	0.89
Sigma power (μV^2)	2.43	1.32	0.62	0.43	0.52	-0.10	-0.22	0.31
**Percent stage 3 sleep**	**53.76**	**22.04**	**14.51**	**<0.001**	**0.001**	**0.02**	0.30	0.16
Percent stage 2 sleep	31.76	18.23	0.82	0.37	0.48	-0.01	-0.13	0.55
**Percent REM sleep**	2.02	6.47	0.00	1.00	1.00	-0.01	**-0.42**	**0.04**
**Sleep disruption index**	**14.81**	**5.75**	**3.98**	**0.046**	0.13	-0.01	-0.38	0.07

Supplemental Table 2: Correlation between participant/sleep metrics and Δrecog. Statistics were computed as described in the previous table.

Measure	Cauchy chi^2	Cauchy *p*	Cauchy *p* (FDR)	OLS r	OLS *p*
Total sleep minutes	0.84	0.36	0.85	0.17	0.40
Stage 2 minutes	0.01	0.93	1.00	-0.20	0.37
**Stage 3 minutes**	**5.56**	**0.02**	0.11	0.22	0.29
REM minutes	0.00	1.00	1.00	-0.39	0.06
**Age** (years)	**15.13**	**<0.001**	**<0.001**	**-0.48**	**0.02**
Number of name cues	0.12	0.73	1.00	0.20	0.36
Minutes between tests	0.25	0.62	1.00	0.22	0.30
Minutes of sleep after last cue	0.70	0.40	0.85	-0.04	0.85
Total stage transitions	2.04	0.15	0.51	0.23	0.29
Sleep fragmentation index	0.07	0.79	1.00	0.19	0.38
Slow oscillation power (μV^2)	0.00	0.95	1.00	0.09	0.67
Delta power (μV^2)	0.87	0.35	0.85	0.15	0.48
**Sigma power** (μV^2)	**10.59**	**0.001**	**0.01**	-0.15	0.50
Percent stage 3 sleep	0.05	0.82	1.00	0.14	0.53
**Percent stage 2 sleep**	**5.02**	**0.03**	0.13	-0.33	0.12
Percent REM sleep	0.00	1.00	1.00	0.38	0.07
Sleep disruption index	0.31	0.58	1.00	-0.20	0.36

Supplemental Table 3: Memory measures (mean ± SEM)**.** Whereas name recall and face recognition scores were not significantly influenced by TMR, the cuing effect was positively correlated with minutes in N3 sleep (for both measures) and negatively correlated with the sleep disruption index (for name recall). These *p* values were computed using robust regression with a Cauchy distribution as described in the methods, and *r* values were calculated from ordinary least squares regression. TMR had a larger influence in participants with more N3 sleep and less sleep disruption.

Mean ± SEM	Pre-sleep		Post-sleep		Cuing effect	Cuing x N3 duration	Cuing x sleep disruption
	Uncued	Cued	Uncued	Cued			
Names recalled	37.08 ±2.50	36.96 ±3.20	37.29 ±1.55	37.71 ±2.94	0.54 ±2.84	***r* = 0.24 *p* = 0.02**	***r* = -0.38 *p* = 0.046**
Names recalled with no hints	23.54 ±2.27	23.46 ±2.32	23.75 ±2.35	22.96 ±2.24	-0.71 ±0.81	*r* = -0.19 *p* = 0.75	*r* = 0.02 *p* = 0.75
Mean hints per name recalled	0.85 ±0.17	0.84 ±0.16	0.87 ±0.18	0.87 ±0.17	-0.01 ±0.07	*r* = -0.03 *p* = 0.43	*r* = -0.09 *p* = 0.87
Recognition d' for faces	2.56 ±0.19	2.58 ±0.18	3.05 ±0.22	2.95 ±0.22	-0.12 ±0.09	***r* = 0.22 *p* = 0.02**	*r* = -0.2 *p* = 0.58

Supplemental Table 4: Differences in sleep and participant measures between the 12 participants with the highest N3 duration and the 12 participants with the lowest N3 duration (median split). Measures are computed as described in Supplemental Table 1.

Measure	High N3	Low N3	*p*
Total sleep minutes	62.75	55.33	0.24
**Stage 2 minutes**	**12.46**	**25.88**	**0.01**
**Stage 3 minutes**	**43.38**	**19.29**	**<0.001**
REM minutes	1.23	1.83	0.77
Age (years)	24.75	22.18	0.18
**Number of cues**	**137.50**	**99.75**	**0.02**
Minutes from presleep test to postsleep test	115.17	111.67	0.57
Minutes of sleep after last cue	33.77	37.54	0.54
**Total stage transitions**	**18.42**	**28.83**	**0.02**
**Sleep fragmentation index**	**17.49**	**32.13**	**<0.001**
**Slow oscillation power** (μV^2)	**721.54**	**308.74**	**<0.001**
**Delta power** (μV^2)	**165.61**	**70.14**	**0.01**
Sigma power (μV^2)	2.56	2.31	0.64
**Percent stage 3 sleep**	**0.71**	**0.36**	**<0.001**
**Percent stage 2 sleep**	**0.18**	**0.45**	**<0.001**
Percent REM sleep	0.02	0.03	0.70
**Sleep disruption index**	**0.12**	**0.18**	**0.01**
