# Supplementary material for: Targeted memory reactivation of face-name learning depends on ample and undisturbed slow-wave sleep
Source: NPJ Sci Learn. 2022 Jan 12;7:1. doi: 10.1038/s41539-021-00119-2 (PMC8755782; doi:10.1038/s41539-021-00119-2)
Supplement: Supplementary file 2 — Reporting Summary [file 41539_2021_119_MOESM2_ESM.pdf]

nature portfolio

Corresponding author(s): Ken Paller

Last updated by author(s): Aug 13, 2021

Reporting Summary

Nature Portfolio wishes to improve the reproducibility of the work that we publish. This form provides structure for consistency and transparency in reporting. For further information on Nature Portfolio policies, see our Editorial Policies and the Editorial Policy Checklist.

Statistics

For all statistical analyses, confirm that the following items are present in the figure legend, table legend, main text, or Methods section.

n/a	Confirmed	
☐	☒	The exact sample size (*n*) for each experimental group/condition, given as a discrete number and unit of measurement
☐	☒	A statement on whether measurements were taken from distinct samples or whether the same sample was measured repeatedly
☐	☒	The statistical test(s) used AND whether they are one- or two-sided<br>*Only common tests should be described solely by name; describe more complex techniques in the Methods section.*
☐	☒	A description of all covariates tested
☐	☒	A description of any assumptions or corrections, such as tests of normality and adjustment for multiple comparisons
☐	☒	A full description of the statistical parameters including central tendency (e.g. means) or other basic estimates (e.g. regression coefficient) AND variation (e.g. standard deviation) or associated estimates of uncertainty (e.g. confidence intervals)
☐	☒	For null hypothesis testing, the test statistic (e.g. *F*, *t*, *r*) with confidence intervals, effect sizes, degrees of freedom and *P* value noted<br>*Give P values as exact values whenever suitable.*
☒	☐	For Bayesian analysis, information on the choice of priors and Markov chain Monte Carlo settings
☒	☐	For hierarchical and complex designs, identification of the appropriate level for tests and full reporting of outcomes
☐	☒	Estimates of effect sizes (e.g. Cohen's *d*, Pearson's *r*), indicating how they were calculated

Our web collection on statistics for biologists contains articles on many of the points above.

Software and code

Policy information about availability of computer code

Data collection	Neurobehavioral Systems Presentation 17.2 for presenting sounds during sleep<br>Psychopy v 1.85.2 for learning procedure and memory tests<br>Custom python code for randomizing faces, names, and class assignments for each participant
Data analysis	JMP 15 (statistical analysis)<br>Graphpad QuickCalc (statistical analysis)<br>MATLAB 2014B<br>EEGLAB 14.1.1b (EEG signal processing)<br>SleepSMG (sleep scoring)<br>Custom MATLAB scripts for calculating sleep disruption index and batch processing of EEG data<br>Custom Python scripts for calculating memory performance measures

For manuscripts utilizing custom algorithms or software that are central to the research but not yet described in published literature, software must be made available to editors and reviewers. We strongly encourage code deposition in a community repository (e.g. GitHub). See the Nature Portfolio guidelines for submitting code & software for further information.

Data

Policy information about availability of data

All manuscripts must include a data availability statement. This statement should provide the following information, where applicable:

- Accession codes, unique identifiers, or web links for publicly available datasets
- A description of any restrictions on data availability
- For clinical datasets or third party data, please ensure that the statement adheres to our policy

The data that support the findings of this study are available from the corresponding author upon reasonable request.

Field-specific reporting

Please select the one below that is the best fit for your research. If you are not sure, read the appropriate sections before making your selection.

☐ Life sciences ☒ Behavioural & social sciences ☐ Ecological, evolutionary & environmental sciences

For a reference copy of the document with all sections, see nature.com/documents/nr-reporting-summary-flat.pdf

Behavioural & social sciences study design

All studies must disclose on these points even when the disclosure is negative.

Study description	This is a quantitative study examining the effect of cues presented during sleep on recall and recognition memory for faces
Research sample	We analyzed data from 24 participants (8 male, 16 female, ages 18-31) who were adults living in the Evanston area. The participant sample was not intended to be a representative sample of the community. Participant eligibility criteria were (1) age 18-35, (2) right-handed, (3) normal or corrected-to-normal vision, (4) People who believed that they could successfully nap in the lab
Sampling strategy	Participants were recruited using fliers placed on campus and social media advertisments.
Data collection	Participants completed all tasks in a a soundproof room using a computer. Tasks were coded in NBS Presentation. EEG , EOG, and EMG data was acquired using a BioSemi ActiveTwo system with 32 scalp electrodes and four EOG/EMG electrodes using stanard AASM procedures.
Timing	Data were collected from September 12, 2016 to December 3, 2016 and from August 21, 2017 to October 26,2017
Data exclusions	We excluded 13 participants with insufficient N3 sleep to deliver all 20 spoken name cues (planned exclusion criterion). We excluded 1 participant from all analyses due to a technical error (no EEG data) and excluded 1 participant from recognition analysis due to a technical error (recognition test scores were not saved)
Non-participation	No participants dropped out/declined participation
Randomization	Participants were randomly allocated

Reporting for specific materials, systems and methods

We require information from authors about some types of materials, experimental systems and methods used in many studies. Here, indicate whether each material, system or method listed is relevant to your study. If you are not sure if a list item applies to your research, read the appropriate section before selecting a response.

Materials & experimental systems

n/a	Involved in the study	
☒	☐	Antibodies
☒	☐	Eukaryotic cell lines
☒	☐	Palaeontology and archaeology
☒	☐	Animals and other organisms
☐	☒	Human research participants
☒	☐	Clinical data
☒	☐	Dual use research of concern

Methods

n/a	Involved in the study	
☒	☐	ChIP-seq
☒	☐	Flow cytometry
☒	☐	MRI-based neuroimaging

Human research participants

Policy information about studies involving human research participants

Population characteristics	See above
Recruitment	Participants were recruited through fliers and social media advertisements. We selected for participants who stated they could likely nap in the afternoon. This is unlikely to affect the results as subjects who did could nap were excluded from analysis. As fliers were placed on campus, a large number of subjects were affiliated with Northwestern University but we do not have strong reason to believe that influenced the effects we observed.
Ethics oversight	Northwestern University IRB

Note that full information on the approval of the study protocol must also be provided in the manuscript.
